# Layered polymeric nitrogen in RbN_3_ at high pressures

**DOI:** 10.1038/srep16677

**Published:** 2015-11-13

**Authors:** Xiaoli Wang, Jianfu Li, Ning Xu, Hongyang Zhu, Ziyu Hu, Li Chen

**Affiliations:** 1 Institute of Condensed Matter Physics, Linyi University, Linyi 276005, P. R. China; 2 Beijing Computational Science Research Center, Beijing, 100084, P. R. China; 3 School of science, Linyi University, Linyi 276005, P. R. China; 4 Department of Physics, Yancheng Institute of Technology, Yancheng 224051, China.; 5 State Key Laboratory of Superhard Materials, College of Physics, Jilin University, Changchun 130012, P. R. China

## Abstract

The structural evolutionary behaviors of nitrogen in RbN_3_ have been studied up to 300 GPa using a particle swarm optimization structure searching method combined with density functional calculations. Three stable new phases with *P*-1, *P*6/*mmm* and *C*2/*m* structure at pressure of 30, 50 and 200 GPa are identified for the first time. The analysis of the crystal structures of three new predicated phases reveals that the transition of N_3_− ions goes from linear molecules to polymeric chains, benzene-like rings and then to polymeric layers induced by pressure. The electronic structures of three predicted phases reveal that the structural changes are accompanied and driven by the change of orbital hybridization of N atoms from *sp* to *sp*^2^ and finally to partial *sp*^3^. Most interestingly, the Rb atoms show obvious transition metal-like properties through the occupation of 4*d* orbitals in high-pressure phases. Moreover, the Rb atoms are characterized by strong hybridization between 4*d* orbitals of Rb and 2*p* orbitals of N in *C*2/*m* structure. Our studies complete the structural evolution of RbN_3_ under pressure and reveal for the first time that the Rb atoms in rubidium nitride possess transition element-like properties under pressure.

Metal azides have been the subjects of many studies including their structural stability, lattice dynamics, electronic structure, and many other physical properties because of their linear azide anion^1^, as well as their significant industrial importance as gas generators and explosives^2^. Recently, taking metal azides as starting materials to synthesis polymeric nitrogen, a potential high-energy-density-material, has become a new topic due to the potential lower synthesis pressure compared with pure nitrogen gas. In nitrogen gas, nitrogen exits in N_2_ molecules and the connection between nitrogen atoms is triple bonds N≡N. In metal azides, nitrogen exits in 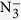 anions and the nitrogen atoms are connected through double bonds N = N. It is expected that the 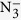 anions in metal azides may form a polymeric nitrogen network more readily than N_2_ molecules, since the N = N have a much lower bonding energy (418 KJ/mol) than the N≡N (954  KJ/mol). Under pressure, 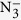 anion will undergo a series of structural transitions accompanied by the change of hybridization type of nitrogen atoms, as shown in Fig. 1. Under low pressure, usually fewer than 30 GPa, 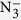 anions maintains their linear structures with *sp* hybridization, in which the crystal maybe undergo orientational phase transition of 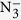 anions induced by pressure^3^,^4^,^5^,^6^,^7^. As pressure increase, 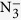 anions translates to a so called pseudo-benzene N_6_ ring with *sp*^2^ hybridization^4^,^5^,^8^,^9^. Continuing to increase pressure, nitrogen will form polymeric structure with partial *sp*^3^ hybridization. For CsN_3_, our previous work indicates that 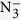 anions will translate to a chain like structure at 51 GPa instead of N_6_ ring^10^. In the process of nitrogen structural transition, alkali metal atoms in azides act as electronic donors to change the connection between nitrogen atoms and electronic properties of compounds. A lot of experimental and theoretical work has been done to study the high-pressure behaviors of nitrogen in LiN_3_^4^,^8^,^11^,^12^,^13^,^14^,^15^, NaN_3_^3^,^5^,^16^, KN_3_^7^,^9^,^14^,^17^,^18^,^19^,^20^,^21^, and CsN_3_^10^,^22^. Therefore, a study of the high-pressure behavior of RbN_3_ would provide more insights into the mechanism of pressure-induced structural evolution of 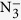 anions. It is helpful to investigate theoretically the pressure effect on rubidium azide and the role of rubidium atoms in the structural evolution process of 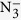 anions.

Under ambient conditions, α-RbN_3_ has the lowest energy with a body-centered tetragonal (bct) lattice of space group *I*4/*mcm*, in which rubidium (Rb), nitrogen 1 (N1), and nitrogen 2 (N2) atoms are located on the 4a,4d, and 8 h Wyckoff positions, respectively (Fig. 2a),which is isostructural to the low-temperature phase of KN_3_ and CsN_3_ in all respects^23^. The nitrogen, linear and symmetric, occupy alternately [0,1,1] and [1 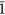 0] directed position in the crystal forming planes ((0 0 1) and (0 0 2) planes) separated by layers of Rb ions. Recently, for the high-pressure behaviors of RbN_3_, we present the *in-situ* X-ray diffraction studies of RbN_3_ up to 42.0 GPa at room temperature^6^. Two pressure-induced orientational phase transitions of α-RbN_3_ (*I*4/*mcm*) → γ-RbN_3_ (*C*2/*m*) → δ-RbN_3_ were identified at 6.5 and 16.0 GPa, respectively.

In this work, we will focus on the structural evolution of anions under high pressure. A series of phase transitions in which 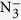 is converted to a chain like structure N_6_ ring and layered polymeric nitrogen have been discovered up to 300 G Pa by using a specifically developed particle swarm optimization (PSO) algorithm technique for crystal structure prediction^24^. To confirm the thermal dynamic stabilities of new phases, phonon dispersion spectroscopies have been calculated. The electronic properties calculations indicate the 4*d* orbitals of Rb have been partially occupied in high-pressure phases and a strong hybridization has been formed between 4*d* orbital of Rb and 2*p* orbital of N in layered structure of RbN_3_.

## Computational Details

We have performed extensive structure searches to uncover the high-pressure structures of RbN_3_ based on a global minimization of free-energy surfaces merging *ab initio* total energy calculations via PSO technique, as implemented in the Crystal Structure Analysis by Particle Swarm Optimization (CALYPSO) code^24^,^25^. This method has successfully predicted the ground state structure for various systems including Nitrogen^26^, Caesium^27^, and superhard carbon nitride^28^. The underlying *ab* initio structural relaxations and electronic band structure calculations are performed in the framework of density functional theory within generalized gradient approximation Perdew-Burke-Ernzerhof (GGA-PBE)^29^, as implemented in the VASP code^30^. The projector augmented wave (PAW)^31^ pseudopotentials are adopted with the PAW potentials taken from the VASP library where 4*p*^6^5*s*^1^ and 2*s*^2^2*p*^3^ are treated as valence electrons for Rb and N atoms, respectively. The cutoff energy (800 eV) for the expansion of the wave function into plane waves and Monkhorst-Pack^32^ *k*-meshes (*k*-points density 0.03 Å^−1^) are chosen to ensure that all the enthalpy calculations are well converged to better than 1 meV/atom. The calculations of net charge are based on Bader analysis^33^,^34^. The phonon calculations are carried out by using a supercell approach as implemented in the PHONOPY code^35^.

## Results and Discussion

**Figure 2:**
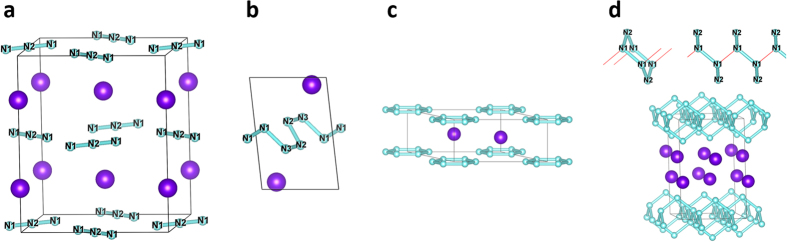
Crystal structures. The ambient condition and the predicted high-pressure phase: (**a**) *I*4/*mcm*; (**b**) *P*-1; (**c**) *P*6/*mmm*; and (**d**) *C*2/*m*. The large and small spheres denote rubidium and nitrogen atoms, respectively. The red lines denote the connection between N_6_ rings in up section of (**d**). The nitrogen atoms in (**a,b,d**) have been labeled N1, N2, and N3 according to their Wyckoff positions.

The variable-cell high-pressure structure predictions have been performed within a pressure region from 0 to 300 GPa, with system containing from one to eight formula units per simulation cell as implemented in CALYPSO code. Our structural searches identified not only the ambient conditions phase *I*4/*mcm* shown in Fig. 2a, three new structures are also depicted in Fig. 2b,d. The lattice constants of predicted structure at ambient pressure *a* = 6.2871 Å and *c* = 7.5106 Å are in agreement with the results obtained in experiment (*a* = 6.3098 Å and *c* = 7.5188 Å^23^) which validates our computational method adopted here. The 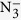 ions are linear and symmetric, and the bond length of N = N is 1.187 Å, which is same as that in potassium azides and well in agreement with the experimental results (1.176 Å^23^). The calculated atomic fractional coordinates are summarized in Table 1. The results indicate that 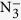 anions undergo transition of 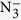 → N chain → N_6_ ring → layered N. Different form converting directly into N_6_ ring structure in LiN_3_^4^,^8^, NaN_3_^5^, and KN_3_^7^,^9^, 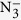 anions translate to chain-like structure, which appears in CsN_3_^10^ at 51 GPa, before entering N_6_ ring structure. The layered nitrogen in *C*2/*m* phase is constructed by chair-like N_6_ rings, as shown in up section of Fig. 2d, completely different from the high-pressure structures in LiN_3_^4^, NaN_3_^5^, and KN_3_^7^. In *C*2/*m* phase, nitrogen atoms have two nonequivalent sites 8j and 4i, respectively. The nitrogen atom located on 8j site is connected with three neighboring N through three N-N bonds and the N located on 4i site is connected with two neighboring N through two N-N bonds.

**Figure 3:**
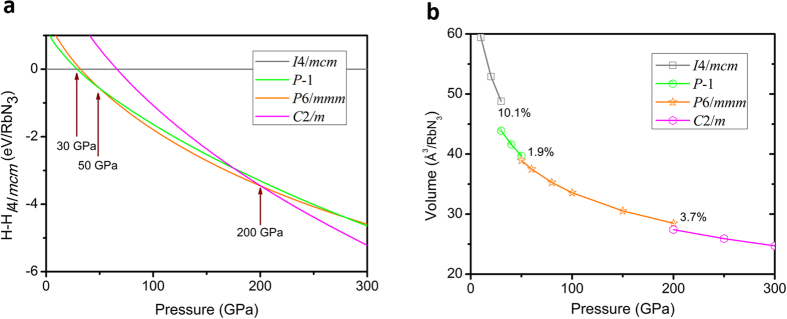
(**a**) Enthalpy of formation of selected structures of RbN_3_ as a function of pressure (relative to the *I*4/*mcm* phase). (**b**) Phase diagram of RbN_3_ at pressure region from 0 to 300 GPa.

To investigate the energetic stabilities of RbN_3_ compound under high pressure, we calculate the formation enthalpy relative to the *I*4/*mcm* structure of RbN_3_ in a pressure range from 0 to 300 GPa, as shown in Fig. 3a. The most stable structure is a tetragonal phase with *I*4/*mcm* symmetry from ambient pressure which is then replaced by a lower-enthalpy *P*-1 structure at 30 GPa. Above 50 GPa, a hexagonal structure with *P*6/*mmm* symmetry is favored over other structures and remains the lowest-enthalpy phase up to 200 GPa. Continuously increasing pressure, the RbN_3_ will translate to a monoclinic structure with *C*2/*m* symmetry. Thorough structure searches using CALYPSO do not find any other structural change up to 300 GPa. The fact that the four structures are in entirely different crystal symmetry suggests that the transitions between them are first order which is indeed confirmed by the calculated P-V curves (Fig. 3b). The reductions of the volumes are found to be 10.1%, 1.9% and 3.7% for the transitions from *I*4/*mcm* to *P*-1, from *P*-1 to *P*6/*mmm* and from *P*6/*mmm* to *C*2/*m*, respectively.

**Figure 4:**
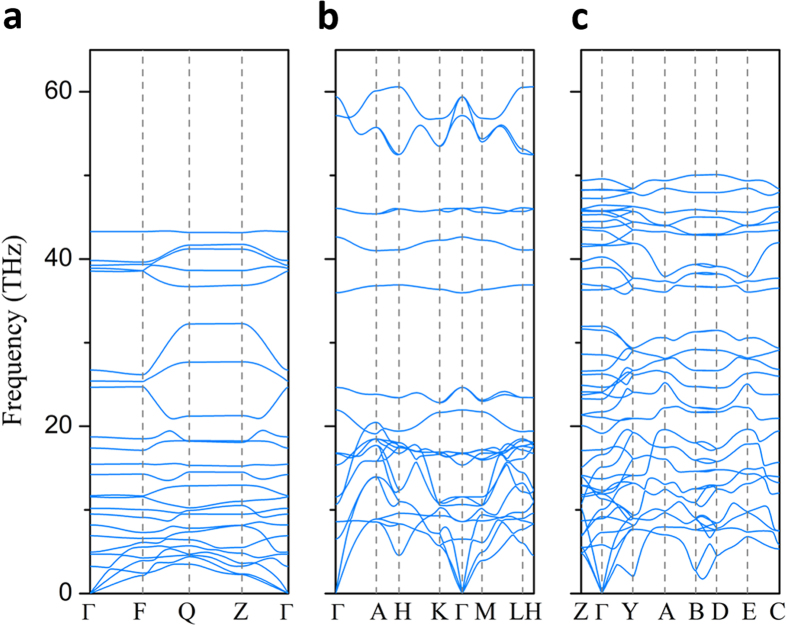
Phonon-dispersion curves of predicted structures. (**a**) *P*-1, (**b**) P6/*mmm*, and (**c**) *C*2/*m* at 40 GPa, 100 GPa and 300 GPa, *respectively*.

The dynamic stability of three predicted structures are examined by calculating the phonon spectra using the supercell method^35^. No imaginary phonon frequency is found in the whole Brillouin zone at the pressure 40 GPa, 100 GPa and 300 GPa, respectively, as shown in Fig. 4.

Under pressure, the structural evolution of the rubidium azide is accompanied by the change of the electronic properties. To explore that, we calculated the electronic structures and their dependence on pressure in several aspects, including the electron localized functions (ELF), band structures, electronic band structure, projected density of states (PDOS), and net charge of Rb atoms.

**Figure 5:**
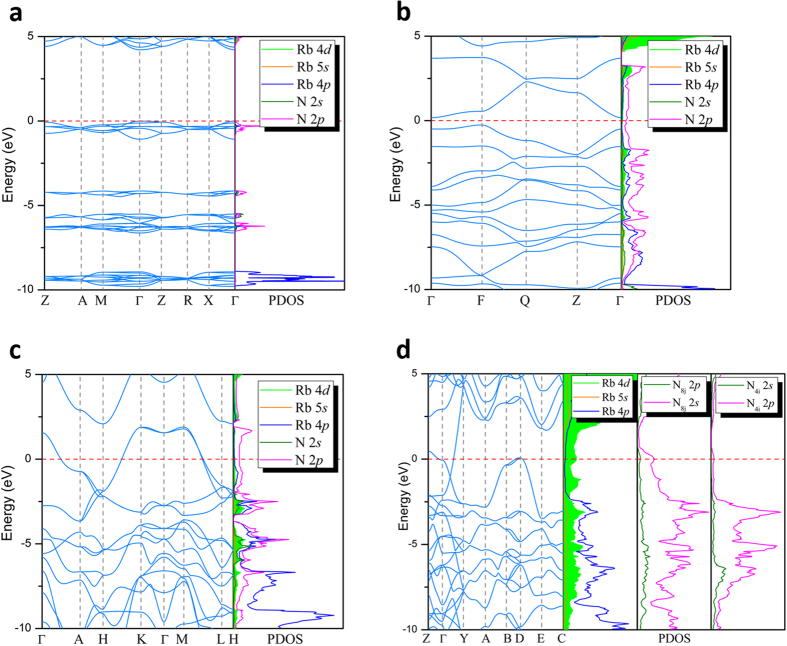
Band structure and projected density of states. (**a**) *I*4/*mcm*, (**b**) *P*-1, (**c**) *P*6/*mmm*, and (**d**) *C*2/*m* at 0 GPa, 40 GPa, 100 GPa and 300 GPa, respectively. Dash line denotes Fermi energy level.

**Figure 6:**
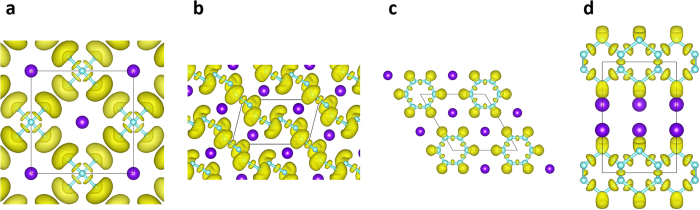
The electron localized functions. (**a**) *I*4/*mcm*, (**b**) *P*-1, (**c**) *P*6/*mmm*, and (**d**) *C*2/*m* at 0 GPa, 40 GPa, 100 GPa and 300 GPa, respectively. The valve of isosurface is 0.8.

As shown in Fig. 5, the four phase transitions of RbN_3_ are accompanied by insulator-metal-metal-metal transitions. At ambient conditions, the *I*4/*mcm* structure is an insulator characterized by a large energy gap of 4.3 eV that is similar to the atmospheric pressure phases of other alkali metal azides^7^,^10^,^12^. However, the *P*-1, *P*6/*mmm* and *C*2/m structures exhibit clear metallic behaviors by evidence of cross of band structures and the finite electronic DOS at the Fermi level. As can be seen from the partial DOS, N-2*p* states contribute most to the valance band and the DOS near the Fermi level in *P*-1 and *P*6/*mmm* structures. The detailed analysis for metallicity of *P*-1 and *P*6/*mmm* can be found in previous works for CsN_3_^10^ and KN_3_^7^,^9^ due the similar electronic properties. For four considered structures, the strong covalent bonding between nitrogen atoms as well as the lone pairs electrons are revealed clearly by the ELF shown in Fig. 6. As pressure increase, the hybridization type between nitrogen atoms undergoes from *sp* (within *I*4/*mcm*) to *sp*^2^ (within *P*-1 and *P*6/*mmm*), then to partial *sp*^3^ (within *C*2/*m*).

**Figure 7:**
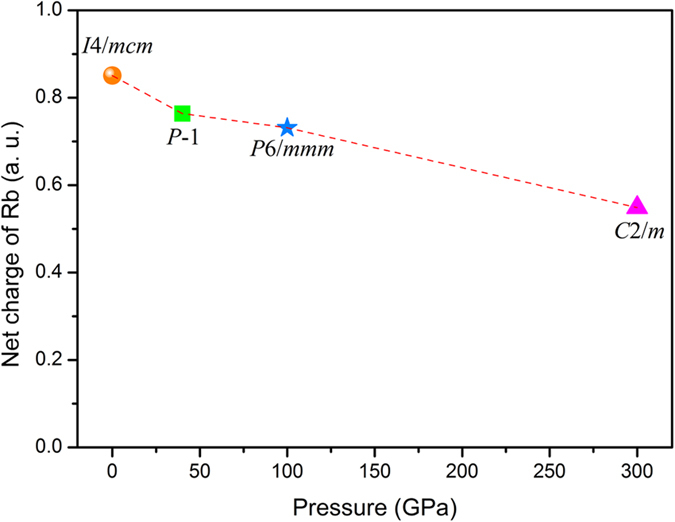
The net charge of Rb atoms based on Bader charge analysis for Rb in *I*4/*mcm*, *P*-1, *P*6/*mmm* and *C*2/*m* phases at 0 GPa, 40 GPa, 100 GPa and 300 GPa, respectively.

More interestingly, the 4*d* orbital of rubidium is partially occupied in high-pressure phases as shown in Fig. 5b,d and there is an obvious orbital hybridization between the 4*d* of Rb and 2*p* of N located on 8j sites in *C*2/*m* phase. To further confirm this result, we recalculate the PDOS of the four phases using a much small Wigner-Seitz radius, in which we change the spherical radius from defaults value in pseudo-potential (2.418 and 0.741 Å for Rb and N) to smaller values based on the Bader analysis, as shown in supplementary Figure S1-S3. The recalculated results are similar to the previous PDOS for the four phases except the valve of densities. To study the impact of partial occupation of 4*d* orbital on electronic properties, we calculate the net charge of Rb atom based on Bader analysis, as shown in Fig. 7. At ambient conditions, the Rb atoms contribute almost one electron (0.85) to three N atoms forming 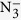 anion.

Under high pressure, the net charge decreases due to the partial occupation of 5*d* orbital, though the Rb atom still loses its 5*s* electron. Though the transition metal-like property of Rb elements has been reported by both experimental and theoretical works in alkali metal elements under high pressure^36^,^37^,^38^, there has been no research addressing that in chemical compounds. The difference that comes from the occupation of *d* orbitals is caused by the *spd* hybridization in alkali metal elements, for RbN_3_ that results from the hybridization between 4*d* of Rb and 2*p* of N which may enhance the stability of compound.

## Conclusion

In summary, we studied the evolution of the structures of RN_3_ under high pressure by using an unbiased automatic structure search method based on first-principles total energy calculations and geometry optimization. We predicted three new high-pressure structures of RbN_3_ with *P*-1, *P*6/*mmm* and *C*2/*m* structure at pressure of 30, 50 and 200 GPa. This result extends the high-pressure structures RbN_3_. The analysis of the electronic structure reveals that the transition trend of 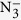 ions from linear molecules to polymer chains, then to benzene-like rings, and finally to layered polymeric nitrogen is driven by the hybridization of N atoms in which the nitrogen hybridized types are *sp*, *sp*^2^, *sp*^2^, and partial *sp*^3^, respectively. For the first time, we reveal that the Rb atoms in RbN_3_ possess obvious transition metal-like properties under high pressure. In *C*2/*m* phase, a strong hybridization has been found between 4*d* orbitals of Rb and 2*p* orbitals of N.

## Additional Information

**How to cite this article**: Wang, X. *et al.* Layered polymeric nitrogen in RbN_3_ at high pressures. *Sci. Rep.* **5**, 16677; doi: 10.1038/srep16677 (2015).

## Supplementary Material

Supplementary Information

## Figures and Tables

**Figure 1:**
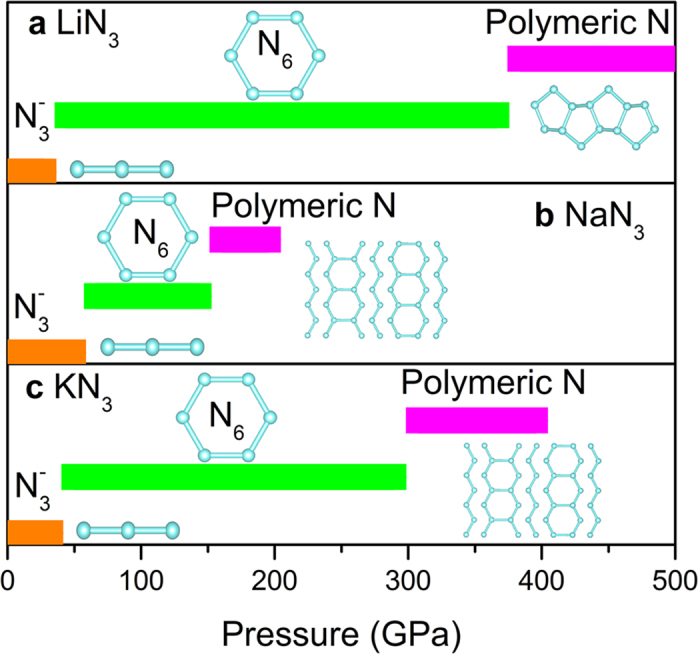
Structural evolution of N_3_^-^ anion in alkali metal azides under compression. (**a**) LiN_3_, (**b**) NaN_3_, and (**c**) KN_3_. Transition pressure and structures are taken from reference LiN_3_^4^, NaN_3_^5^, and KN_3_^7^, respectively.

**Table 1:** The unit-cell parameters and atomic positions of the *I*4/*mcm*, *P*-1, *P*6/*mmm* and *C*2/*m* phase at 0 GPa, 40 GPa, 100 GPa and 300 GPa, respectively.

Space group	Pressure (GPa)	Lattice parameters (Å, ˚)		Atom	Wyckoff positions	x	y	z
*I*4/*mcm*	0	a = 6.2871 (6.3098)	α = 90	Rb	4a	0	0	0.25
		b = 6.2871 (6.3098)	β = 90	N1	8h	0.3664	0.1336	0
		c = 7.5106 (7.5188)	γ = 90	N2	4d	0.5	0	0
*P*-1	40	a = 3.3263	α = 96.7544	Rb	2i	0.8007	0.9350	0.7555
		b = 4.7467	β = 75.1448	N1	2i	0.1171	0.5467	0.8935
		c = 5.5230	γ = 97.8611	N2	2i	0.4330	0.6051	0.4513
				N3	2i	0.6319	0.6399	0.2184
*P*6/*mmm*	100	a = 5.5482	α = 90	Rb	2d	0.3333	0.6667	0.5
		b = 5.5482	β = 90	N	6j	0.7684	0.7684	0
		c = 5.5482	γ = 120					
*C*2/*m*	300	a = 3.9946	α = 90	Rb	4i	0.2234	0	0.3843
		b = 4.0911	β = 98.6538	N1	8j	0.8612	0.2517	0.9327
		c = 6.1675	γ = 90	N2	4i	0.8201	0.5	0.8029
