# Supplementary material for: Layered polymeric nitrogen in RbN3 at high pressures
Source: Sci Rep. 2015 Nov 13;5:16677. doi: 10.1038/srep16677 (PMC4643253; doi:10.1038/srep16677)

**Supplementary information**

**Layered polymeric nitrogen in RbN_3_ at high pressures**

Xiaoli Wang^1,2^, Jianfu Li^3*^, Ning Xu^4^, Hongyang Zhu^5^, Ziyu Hu^2^ and Li Chen^1^

^1^Institute of Condensed Matter Physics, Linyi University, Linyi 276005, P. R. China

^2^Beijing Computational Science Research Center, Beijing, 100084, P. R. China

^3^School of science, Linyi University, Linyi 276005, P. R. China

^4^Department of Physics, Yancheng Institute of Technology, Yancheng 224051, China.

^5^State Key Laboratory of Superhard Materials, College of Physics, Jilin University, Changchun 130012, P. R. China

**Figure S1** | The projected density of states for *P*-1 at 40GPa. Dash line denotes Fermi energy level. Due to the dramatically changing in atomic volume under high pressure, we change the spherical radius according to the Bader analysis to get a more accurate PDOS. The radius of rubidium and nitrogen atoms are equal to 1.2816 Å and 0.6110 Å, respectively.


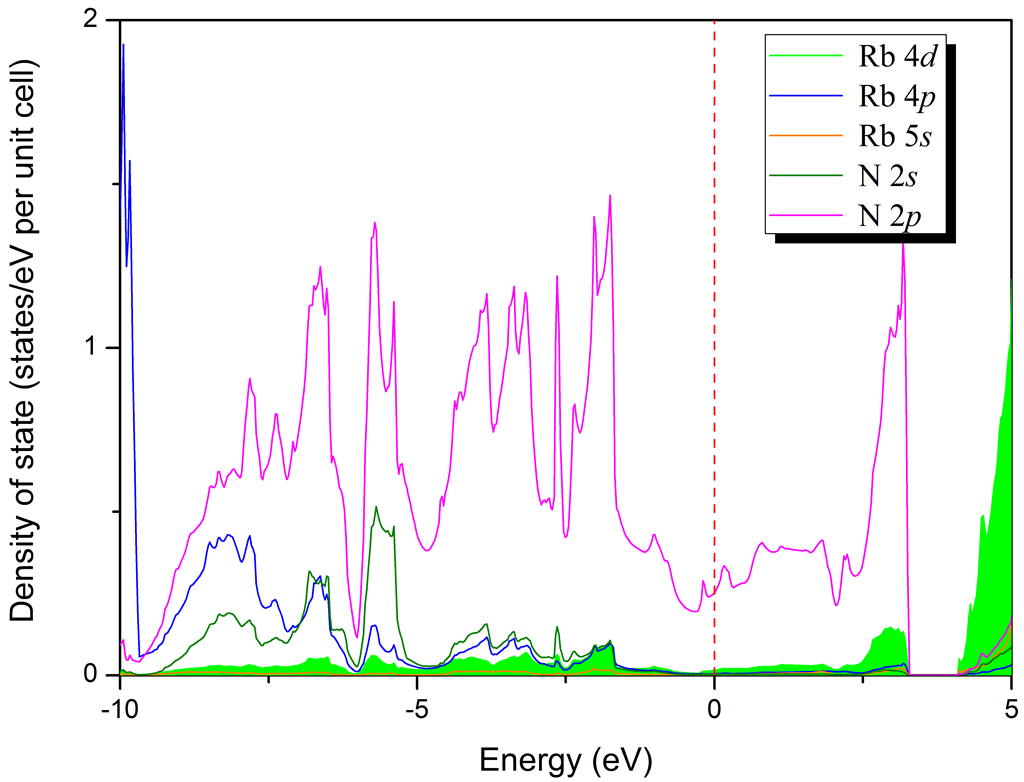


**Figure S2** | The projected density of states for *P*6/*mmm* at 100 GPa. The radius of rubidium and nitrogen atoms are equal to 1.3031 Å and 0.6025 Å, respectively.


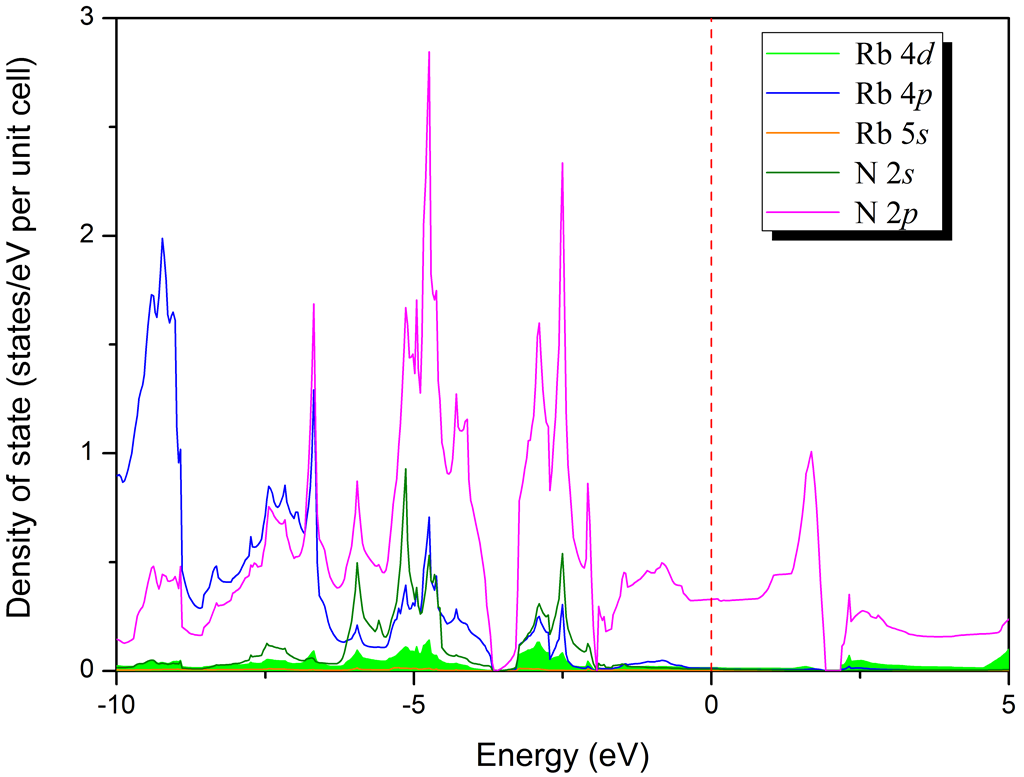


**Figure S3** | The projected density of states for *C*2/*m* at 300GPa. The radius of rubidium and nitrogen atoms are equal to 1.1331 Å and 0.5784 Å, respectively.


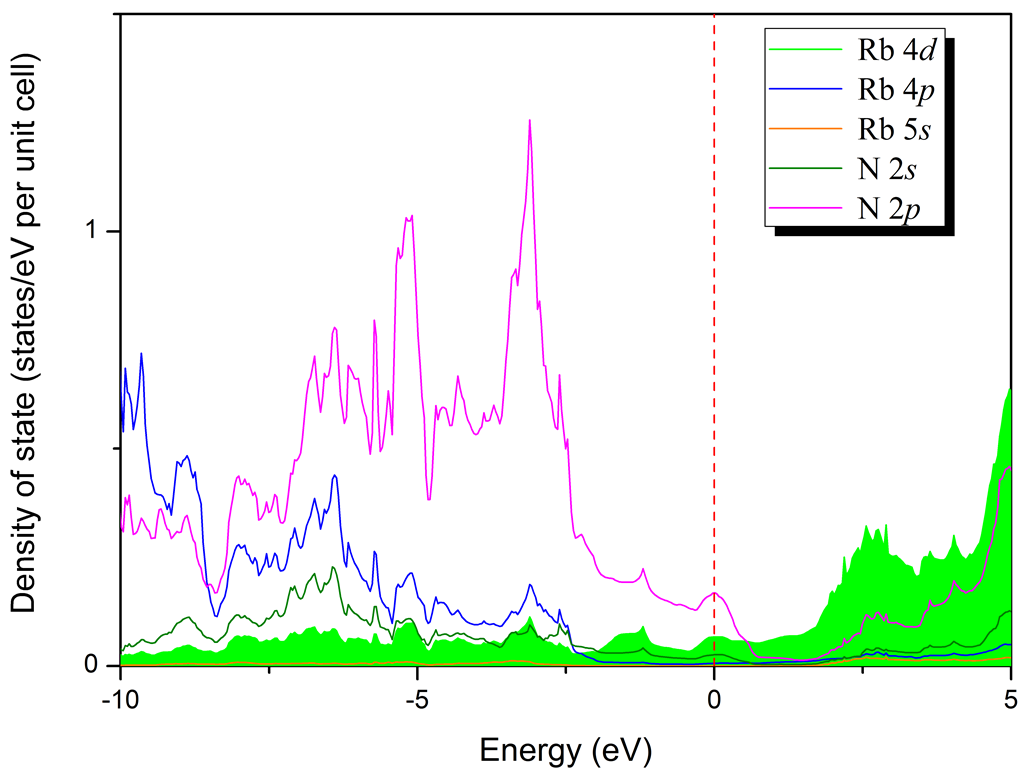

Supplement: Supplementary Information [file srep16677-s1.doc]
